# Impact of *DYRK1A* Expression on *TNNT2* Splicing and Daunorubicin Toxicity in Human iPSC-Derived Cardiomyocytes

**DOI:** 10.1007/s12012-022-09746-6

**Published:** 2022-05-21

**Authors:** Romina Beatriz Cejas, Miriam Tamaño-Blanco, John Edgar Fontecha, Javier Guillermo Blanco

**Affiliations:** 1 Department of Pharmaceutical Sciences, School of Pharmacy and Pharmaceutical Sciences, The State University of New York at Buffalo, Buffalo, NY 14214 USA; 2 Group for Applied Mathematical Modeling and Analytics (GAMMA), Industrial and Systems Engineering, The State University of New York at Buffalo, Buffalo, NY 14214 USA

**Keywords:** Human cardiomyocytes, Anthracyclines, Alternative splicing, Troponin, Heart, Cardiotoxicity

## Abstract

Cardiac troponin T (encoded by *TNNT2*) is involved in the contraction of cardiomyocytes during beating. The alternative splicing of *TNNT2* results in four transcript variants with differential Ca^2+^ sensitivity. The splicing of *TNNT2* involves phosphorylation of the splicing factor SRSF6 by DYRK1A. Altered *TNNT2* splicing patterns have been identified in failing human hearts. There is a paucity of studies describing *DYRK1A-SRSF6-TNNT2* interplays in human cardiomyocytes. Also, it is not known whether the sensitivity of cardiomyocytes to cardiotoxic anthracyclines is modified in the context of variable *DYRK1A-TNNT2* expression. In this study, we investigated the impact of *DYRK1A* on the endogenous expression of *TNNT2* splicing variants in iPSC-derived cardiomyocytes. We also examined whether *DYRK1A* expression modifies the sensitivity of cardiomyocytes to the cardiotoxic drug daunorubicin (DAU). *DYRK1A* over-expression increased the abundance of *TNNT2* fetal variants by ~ 58% whereas the abundance of the adult *cTnT3* variant decreased by ~ 27%. High *DYRK1A* expression increased the phosphorylation of SRSF6 by ~ 25–65%. DAU cytotoxicity was similar between cardiomyocytes with variable levels of *DYRK1A* expression. *DYRK1A* over-expression ameliorated the impact of DAU on beating frequency. This study lays the foundation to further investigate the contribution of variable *DYRK1A-TNNT2* expression to Ca^2+^ handling and beating in human cardiomyocytes.

**Supplementary Information:**

The online version contains supplementary material available at 10.1007/s12012-022-09746-6.

## Introduction

Cardiomyocytes derived from human-induced pluripotent stem cells (iPSC cardiomyocytes) are an informative platform for the study of various cellular processes. iPSC cardiomyocytes express structural and functional genes that are key to myocardial function. Under specific culture conditions, iPSC cardiomyocytes form a spontaneously beating syncytium [1, 2]. Cardiac troponin T (cTnT), a protein encoded by the *TNNT2* gene, is involved in the contraction of cardiomyocytes during beating. The alternative splicing of *TNNT2* involving exons 4 and 5 results in four *cTnT* transcript variants namely *cTnT1* (exon 5+, exon 4+), *cTnT2* (exon 5+, exon 4−), *cTnT3* (exon 5−, exon 4−) and *cTnT4* (exon 5−, exon 4+). The splicing patterns of the *TNNT2* gene change during embryonic and postnatal heart development. In human heart, *cTnT1* is expressed at high levels in fetal heart, and *cTnT2* is expressed at low levels throughout development. *cTnT3* is the predominant variant in adult heart, and *cTnT4* is expressed in fetal and in failing adult heart [3]. *cTnT3* is the only variant expressed in mouse adult heart, and coexistence of two or more *cTnT* variants in transgenic mice results in decreased myocardial contractility [4]. cTnT isoforms confer differential Ca^2+^ sensitivity to cardiomyocytes. For example, *TNNT2* splicing variants that retain exon 5 (i.e., *cTnT1* and *cTnT2*) are mainly expressed in fetal and neonatal heart and result in myofibrils that are more sensitive to Ca^2+^ as determined by measurements of force development [5, 6]. In the adult heart, the expression of the fetal variants *cTnT1* and *cTnT2* is low or undetectable, and altered patterns of fetal and adult *cTnT* transcript variants have been identified in failing human hearts and in animals with dilated cardiomyopathy [7, 8].

DYRK1A (dual-specificity tyrosine phosphorylation-regulated kinase 1A) is a protein kinase with a wide spectrum of targets [9, 10]. The *DYRK1A* gene is located in chromosome 21. Altered *DYRK1A* gene expression due to trisomy 21 (Down syndrome) has been linked to the pathogenesis of several conditions in Down syndrome including alterations in neuronal development, cognitive defects, heart defects, and hematological malignancies [11]. In cardiomyocytes, DYRK1A participates in cell proliferation and differentiation during fetal and early neonatal development [12]. DYRK1A has been identified as a negative regulator of cardiac hypertrophy through a mechanism involving the calcineurin/nuclear factor of activated T cells (NFAT) signaling pathway [13, 14]. Of note, increased DYRK1A expression in hearts from Ts65Dn mice, a mouse model of Down syndrome, impacts the splicing of *TNNT2* and the relative proportions of *cTnT* transcript variants. In line, *DYRK1A* overexpression in human fetal kidney-derived HEK-293 cells increased the splicing of an artificial *TNNT2* “mini-gene” construct [15]. Phosphorylation of the SR splicing factor SRSF6 by DYRK1A modulates exon inclusion in *TNNT2* transcript variants [15]. Comparative analysis of the expression of the *DYRK1A-SRSF6-TNNT2* pathway in myocardial tissue from individuals with and without Down syndrome revealed increased levels of phosphorylated SRSF6 and ~ 50% higher expression of fetal *TNNT2* transcript variants in trisomic myocardium [16]. Whereas the role of DYRK1A during the alternative splicing of *TNNT2* is becoming evident, there is still a paucity of data derived from observations in beating human cardiomyocytes.

Patients treated with anthracycline-based chemotherapeutic regimens may develop serious adverse side-effects, including cardiotoxicity. The cardiotoxicity exerted by anthracyclines spans a spectrum of signs and symptoms ranging from perturbations in cardiac rhythm and function to severe cardiomyopathy and congestive heart failure [17–20]. Interindividual variability between anthracycline exposure and cardiotoxic outcomes suggest that genetic factors could contribute to the risk for anthracycline-related cardiotoxicity [21]. The sensitivity of human cardiomyocytes to cardiotoxic anthracyclines in the context of variable *TNNT2* and *DYRK1A* expression remains to be explored.

In this study, we investigated the impact of *DYRK1A* expression on the relative abundance of endogenous *TNNT2* splicing variants in iPSC cardiomyocytes. Interplays between *DYRK1A* expression and SRSF6 phosphorylation were examined by the analysis of SRSF6 phosphorylation in the context of increased *DYRK1A* expression. We also determined whether increased *DYRK1A* expression modifies beating frequency and sensitivity to the cardiotoxic drug daunorubicin. This study contributes new information on the expression of endogenous *TNNT2* transcript variants and daunorubicin-induced cardiotoxicity in the context of altered *DYRK1A* expression in a model of beating human cardiomyocytes.

## Materials and Methods

### Human iPSC Cardiomyocytes

The Institutional Review Board of the State University of New York at Buffalo (UB-IRB) approved this research. UB-IRB determined that this research is not research with human subjects. Human induced pluripotent stem cell-derived cardiomyocytes (iPSC cardiomyocytes) clone 11713 from Fujifilm Cellular Dynamics were used in this study. iPSC cardiomyocytes were cultured according to the provider’s specifications using the recommended plating and maintenance media (iCell Cardiomyocytes Kit, R1117, Fujifilm Cellular Dynamics). iPSC cardiomyocytes were seeded at a density of ~ 63,000 cardiomyocytes/cm^2^ in fibronectin-coated 96-well plates to promote the formation of a beating syncytium. iPSC cardiomyocytes were cultured in standard incubator conditions at 37 °C, 5% CO_2_, and 95% relative humidity for at least 6 days post initial plating.

### Transfections and Drug Treatments

iPSC cardiomyocytes at day 6 post initial plating were transfected with 100 ng plasmid DNA using ViaFect transfection reagent (E4981, Promega) and the following human *DYRK1A* and *SRSF6* expression constructs: DYRK1A human clone (NM_001396, SC314641, Origene), DYRK1A human tagged ORF Clone (NM_001396, RG212584, Origene), and SRSF6 cDNA ORF clone N-HA tag (HG19052-NY, Sinobiological). Control cultures were transfected with ~ 100 ng of empty PCMV6XL5 vector (Origene).

Drug treatments were initiated 24 h post-transfection with expression constructs. Daunorubicin (DAU, 14159, Cayman Chemical) and epigallocatechin gallate (EGCG, 709035, Cayman Chemical) were added to culture media for a total incubation time of 14 h. DMSO vehicle was added to controls. After treatments with DAU, iPSC cardiomyocytes were washed with PBS, and incubated in fresh culture medium, or medium supplemented with EGCG for 24 h. Cell viability was determined with the CellTiter-Glo luminescent viability kit (G7570, Promega).

### Quantitative Real-Time Polymerase Chain Reaction

Total RNA was isolated with Trizol (Thermo Fisher). The expression of *TNNT2* transcript variants (*cTnT1/cTnT2*, *cTnT3*, and *cTnT4*) and *DYRK1A* mRNA was analysed with specific primers (Table S1) [16]. Total RNA (5 ng) was reverse transcribed and amplified with the iTaq Universal SYBR Green One-Step kit (Bio-Rad). *DYRK1A*, *TNNT2* transcript variants, and the reference gene *B2M* were amplified in parallel in a CFX96 Touch Real-Time PCR detection system (Bio-Rad) with the following cycling parameters: 50 °C for 10 min (reverse transcription), 95 °C for 1 min, followed by 44 cycles of 95 °C for 10 s and 60.5 °C for 20 s. Calibration curves were prepared to analyse linearity and PCR efficiency. qRT-PCR data were analysed using the ΔΔ*Ct* method with the CFX manager software (Bio-Rad). The relative abundance of *DYRK1A* and *TNNT2* transcript variants was determined with the Δ*Ct* method. The proportion of *TNNT2* variants was calculated as follows:

$${\text{Proportion of}}\;cTnT_{v} = \frac{{\Delta Ct\;cTnT_{{v\;B2M\;{\text{normalized}}}} }}{{{\text{Sum}}\;\Delta Ct \, \left( {{{cTnT1} \mathord{\left/ {\vphantom {{cTnT1} {cTnT2,cTnT3,cTnT4}}} \right. \kern-\nulldelimiterspace} {cTnT2,cTnT3,cTnT4}}} \right)_{{B2M\;{\text{normalized}}}} }},$$

where *v* represents variants *cTnT1*/*cTnT2*, *cTnT3*, or *cTnT4*.

### Live Cell Imaging and Image Analysis

At day 14 post initial plating, iPSC cardiomyocytes cultured in plates suitable for fluorescence microscopy were incubated with medium supplemented with 1 µM Fluo-4 AM (F14217, Molecular probes) for 30 min at 37 °C. After incubation, Fluo-4 was removed, replaced with fresh media, and cells were maintained in the incubator for up to 30 min before imaging. Cell imaging was performed at 37 °C, 5% CO_2_ in a humidified incubator using a Dragonfly spinning disk confocal microscope (Andor Technology Ltd.) attached to a DMi8 base (Leica Microsystems). Image stacks (16 bits, 0.15 μm per pixel) were obtained by imaging 240 frames in a total interval of 30 s with a Zyla 4.2 PLUS sCMOS camera using a PlanApo 40× 1.10 NA water immersion objective. Images from ~ 10 fields/well were randomly obtained. Independent incubations with Fluo-4 AM were performed in at least three wells for each condition.

Image analysis was performed with the Fiji (ImageJ) software [22]. Comparisons were performed by analyzing similar numbers of cellular regions of interest (ROIs) per condition with identical image processing parameters. ROIs comprised a circular section of 3811 µm^2^ created with the oval selection tool. For cardiomyocytes forming a synchronized contracting syncytium, the number of fluorescence peaks was similar for any ROIs comprised within a given image stack (Fig. S1). Based on this observation, one ROI per field of view was considered for analysis, unless unsynchronized beating was noticed within a given image stack. To quantify the number of beats per ROI, a list of fluorescence values vs time was obtained using the Plot *Z*-axis profile tool in Fiji. Values were saved as text files and exported to Excel 2016 (Microsoft Office) for further analysis. A macro was designed to find and count the minimum and maximum peaks of fluorescence for each ROI during a total time of 30 s. The number of beatings was defined as the total number of maximum peaks detected during 30 s.

### Western Blot and Dot-Blot

Protein samples were denatured with NuPAGE LDS sample buffer containing NuPAGE sample reducing agent and boiled at 70 °C for 10 min prior to use. Proteins were separated by gel electrophoresis using NuPAGE Novex 4–12% Bis-Tris precast gels and transferred onto PVDF membranes using the iBlot gel transfer device (Thermo Fisher Scientific). For dot-blot assays, protein samples were spotted on nitrocellulose membranes (88018, Thermo Fisher Scientific) and dried at room temperature. Membranes were blocked with 5% non-fat milk in 0.2% Tween 20 phosphate buffered saline (PBS) for 30 min at room temperature and probed overnight at 4 °C with the following primary antibodies: mouse monoclonal anti-phosphoepitope SR proteins (1:500, MABE50, Milipore), rabbit anti-HA Tag (1:250, SG77, Invitrogen), mouse monoclonal anti-DYRK1A (1:200, sc-100376, Santa Cruz). Next, membranes were washed and incubated with StarBright Blue 700 goat anti-mouse IgG secondary antibody (1:2500, 12004159, Bio-Rad), StarBright Blue 520 goat anti-rabbit IgG secondary antibody (1:2500, 12005869, Bio-Rad) and hFAB rhodamine anti-tubulin antibody (1:2500, 12004165, Bio-Rad) for 1 h at room temperature. Immunoreactive bands were visualized in a ChemiDoc MP gel imaging system (Bio-Rad). Densitometric analysis was performed using Fiji (ImageJ) software [22].

### Immunoprecipitation

Immunoprecipitation (IP) assays were performed with an anti-HA immunoprecipitation kit (IP0010, Sigma-Aldrich). Briefly, transfected iPSC cardiomyocytes cultured in 24-well plates were lysed in CelLytic M reagent supplemented with protease inhibitor cocktail (Thermo Fisher Scientific) and Halt phosphatase inhibitor (Thermo Fisher Scientific). Cell lysates were incubated overnight at 4 °C with anti-HA-affinity gel in a mini-spin column. After wash, retained proteins were eluted from columns by incubation at 95 °C for 10 min with NuPAGE LDS sample buffer followed by centrifugation. The resulting IP samples were analyzed by Western blot and dot-blot as described above.

### Phosphatase Treatment

Cell lysates from AC16 human cardiomyocytes (SCC109, Sigma-Aldrich) were incubated with 20 units of calf intestine alkaline phosphatase (CIAP, 18009, Invitrogen) in a 20 μl reaction volume containing 50 mM Tris/HCl (pH 9.3) for 1 h at 37 °C. AC16 cells were previously co-transfected with *DYRK1A* and *SRSF6*-HA tag expression constructs, as described above. Samples before and after CIAP treatment were analyzed by immunoblotting.

### Data Processing and Statistical Analysis

Data processing was performed with Excel 2016 (Microsoft Office). Statistical analyses were performed with GraphPad Prism version 9. The D’Agostino & Pearson omnibus normality test was used to determine the normality of data sets. Comparisons between the means of two groups were performed with the Student’s *t* test or Mann–Whitney’s *U* test for sets with normal and non-normal distributions, respectively. All data were expressed as mean ± SD.

## Results

### Expression of TNNT2 Transcript Variants in the Context of DYRK1A Over-Expression

**Fig. 1:**
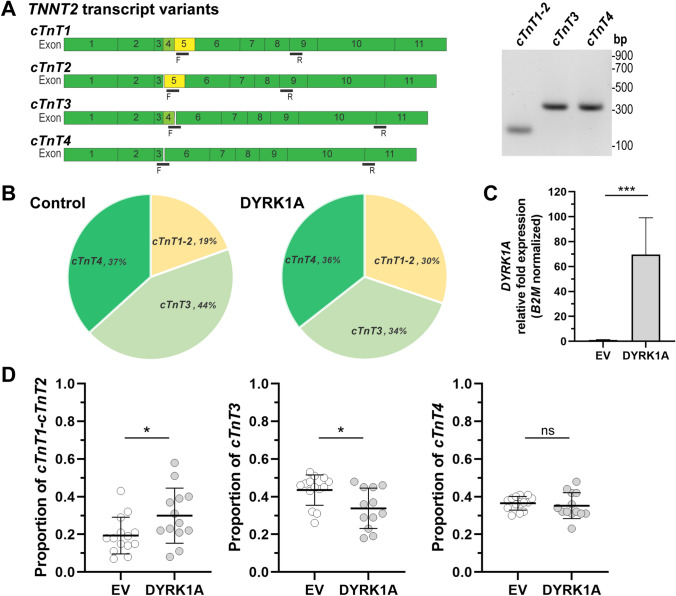
Impact of *DYRK1A* overexpression on the relative abundance of endogenous *TNNT2* transcript variants in iPSC cardiomyocytes. **A** PCR amplification strategy for the analysis of fetal (*cTnT1* and *cTnT2*) and adult (*cTnT3* and *cTnT4*) *TNNT2* transcript variants. F: forward primer, R: reverse primer. **B** Relative abundance of *TNNT2* transcript variants in basal conditions (control) or in cells overexpressing *DYRK1A* (DYRK1A). **C** Relative *DYRK1A* mRNA fold expression in cells transfected with an empty vector (EV, control) or a *DYRK1A* expression construct (DYRK1A). **D** Relative abundance of fetal and adult *TNNT2* transcript variants in basal conditions and in cardiomyocytes overexpressing *DYRK1A*. Each point represents the mean from independent transfections, with two determinations performed in triplicates. Horizontal bars show the mean ± SD. ***P < 0.001, *P < 0.05, *ns* not significant, Student’s *t* test

It is known that increased *DYRK1A* expression impacts the splicing of *TNNT2* and the proportion of *cTnT* transcript variants in hearts from a mouse model of Down syndrome and in human non-cardiac cells [15]. Here, we examined whether *DYRK1A* over-expression modifies the pattern of *TNNT2* splicing in human iPSC cardiomyocytes. The endogenous expression of the fetal variants *cTnT1* and *cTnT2,* and the adult *cTnT3* and *cTnT4* transcript variants was examined with specific PCR primers (Fig. 1A, Table S1). *DYRK1A* over-expression (> 60-fold increase in *DYRK1A* mRNA) caused a ~ 58% increase in the proportion of *cTnT1* and *cTnT2* fetal variants (*cTnT1/2*_*DYRK1A*_: 0.30 ± 0.15, *cTnT1/2*_*EV*_: 0.19 ± 0.10) and a ~ 27% decrease in the proportion of the *cTnT3* variant (*cTnT3*_*DYRK1A*_: 0.34 ± 0.11, *cTnT3*_*EV*_: 0.43 ± 0.08) in comparison to vehicle-transfected controls. There were no significant changes in the proportion of *cTnT4* splicing variants (*cTnT4*_*DYRK1A*_: 0.35 ± 0.07, *cTnT4*_*EV*_: 0.36 ± 0.04) (Fig. 1B–D)*.*

**Fig. 2:**
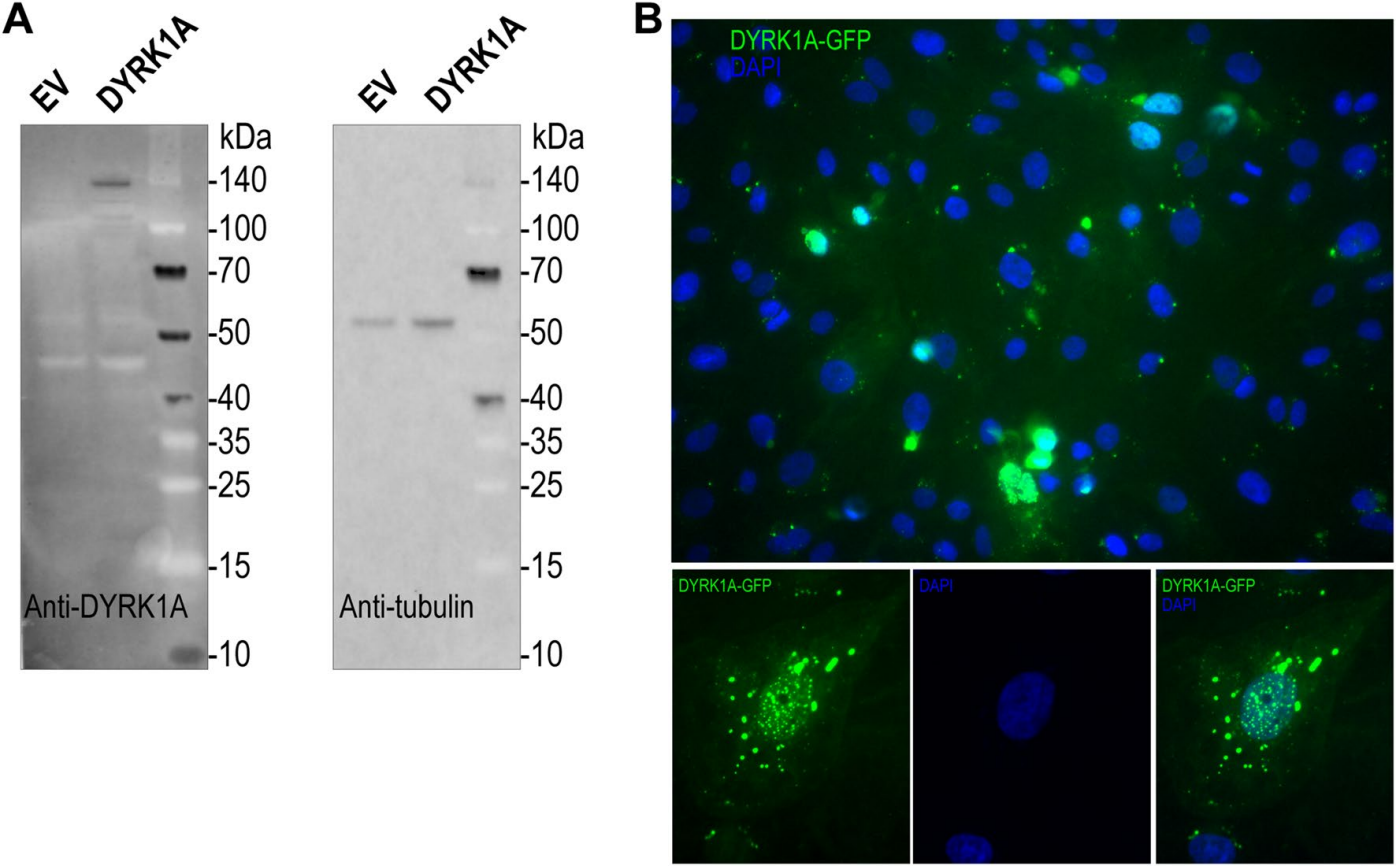
DYRK1A protein expression in iPSC cardiomyocytes. **A** Detection of DYRK1A with an anti-DYRK1A antibody (left panel). β-Tubulin was assayed as loading control (right panel). **B** Representative fluorescence microscopy of a complete field of view (upper panel) and in one individual cell (lower panels) showing the expression and subcellular distribution of DYRK1A-GFP (green). Cells were analyzed 72 h post-transfection with an empty vector (EV) or a DYRK1A-GFP expression construct

The expression of DYRK1A protein was assessed by fluorescence microscopy and immunoblotting in iPSC cardiomyocytes transfected with a *DYRK1A-GFP* expression construct (Fig. 2). The level of DYRK1A protein expression was very low to null in basal conditions (EV), and there was a relatively weak protein signal in DYRK1A-transfected cells (Fig. 2A). Moderate DYRK1A-GPF protein expression was observed by fluorescence microscopy in DYRK1A-transfected cells. DYRK1A was present in the nucleus and cytoplasm of cardiomyocytes. The distribution of DYRK1A showed a dotted pattern resembling nuclear speckles (Fig. 2B).

### SRSF6 Phosphorylation and Expression of TNNT2 Transcript Variants

**Fig. 3:**
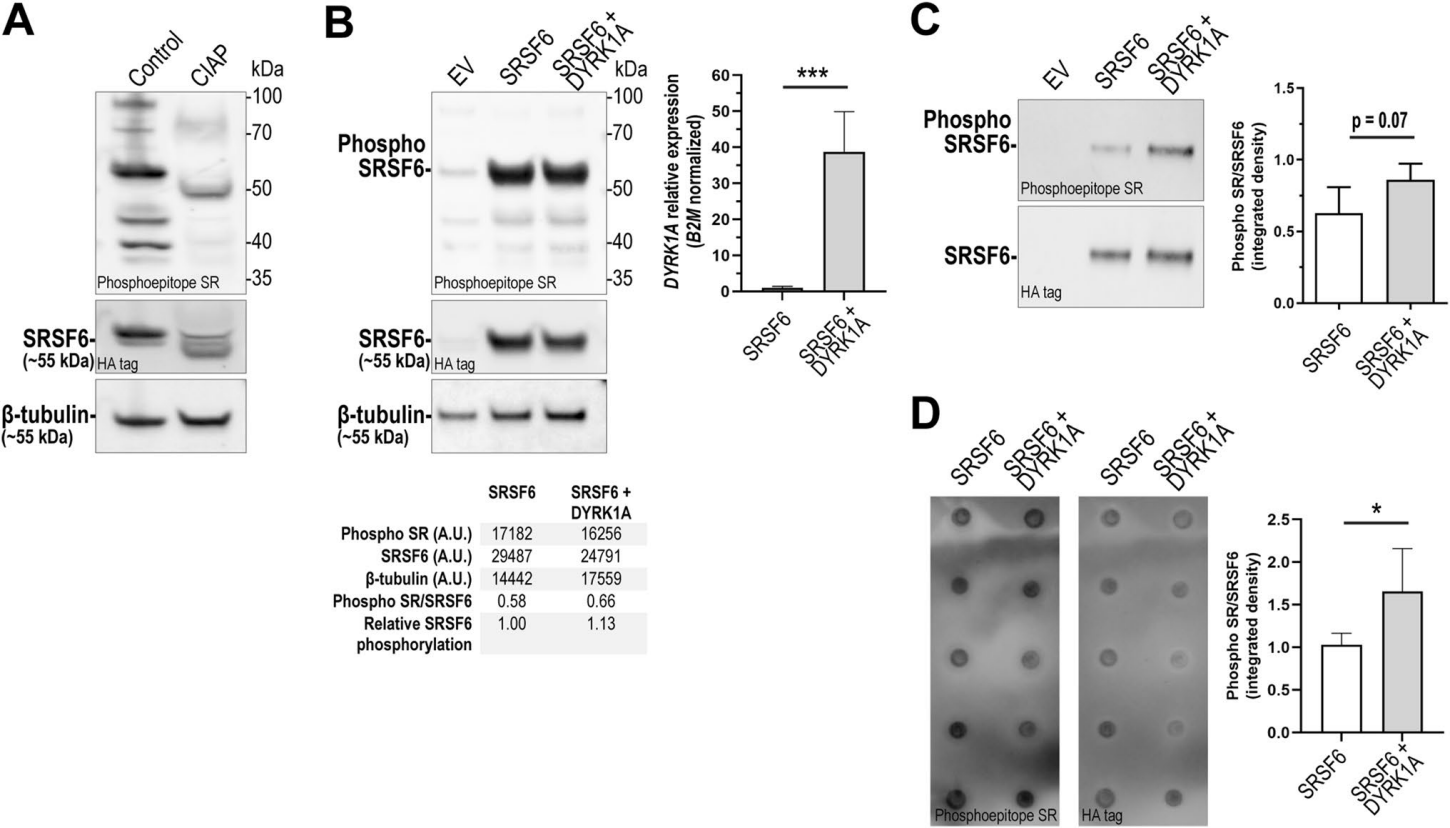
SRSF6 phosphorylation in iPSC cardiomyocytes overexpressing *DYRK1A*. **A** Evaluation of the anti-phosphoepitope SR antibody in total protein extracts from AC16 cardiomyocytes treated with calf intestine alkaline phosphatase (CIAP). **B** Detection of phospho-SRSF6 and SRSF6 in iPSC cardiomyocytes transfected with empty vector (EV), *SRSF6-HA* expression construct (SRSF6) or co-transfected with *DYRK1A* and *SRSF6-HA* (SRSF6 + DYRK1A). β-Tubulin was assayed as loading control. Lower panel: densitometric analysis. Upper right panel: relative *DYRK1A* mRNA fold expression. (A.U.: arbitrary units). **C** Detection of phosphorylated SRSF6 in immunoprecipitated samples from iPSC cardiomyocytes. Right panel: densitometric analysis. Horizontal bars show the mean ± SD from determinations performed in quintuplicate. **D** Dot-blot analysis of phosphorylated SRSF6 in immunoprecipitated samples. Right panel: densitometric analysis. Horizontal bars show the mean ± SD from determinations performed in quintuplicate. ***P < 0.001, *P < 0.05, Student’s *t* test

Phosphorylation of SRSF6 modulates exon inclusion in *TNNT2* transcript variants [15]. To determine the impact of *DYRK1A* overexpression on SRSF6 phosphorylation, we expressed a hemagglutinin-tagged version of SRSF6 (SRSF6-HA). Expression of SRSF6-HA facilitates the detection of phosphorylation by combining HA-protein precipitation plus the antibody for detection of phosphoepitopes in SR proteins. First, we examined the specificity of the antibody that recognizes phosphoepitopes in SR proteins. Immunoblotting analysis of total protein extracts from AC16 cardiomyocytes expressing SRSF6-HA showed a decrease in band intensity in samples treated with calf intestine alkaline phosphatase (CAIP). This observation is consistent with recognition of SR phosphoepitopes by the anti-SR antibody (Fig. 3A). In iPSC cardiomyocytes overexpressing SRSF6, there was a strong signal at ~ 55 kDa consistent with detection of SRSF6-HA in cellular lysates and HA-immunoprecipitates (Fig. 3B, C). In the context of *DYRK1A* overexpression (*DYRK1A* mRNA expression > 39-fold), there was a trend towards ~ 25% increase in the amount of phosphorylated SRSF6 as determined by Western blotting and a ~ 65% increase as determined by dot-blot (Figs. 3C, D, S2).

**Fig. 4:**
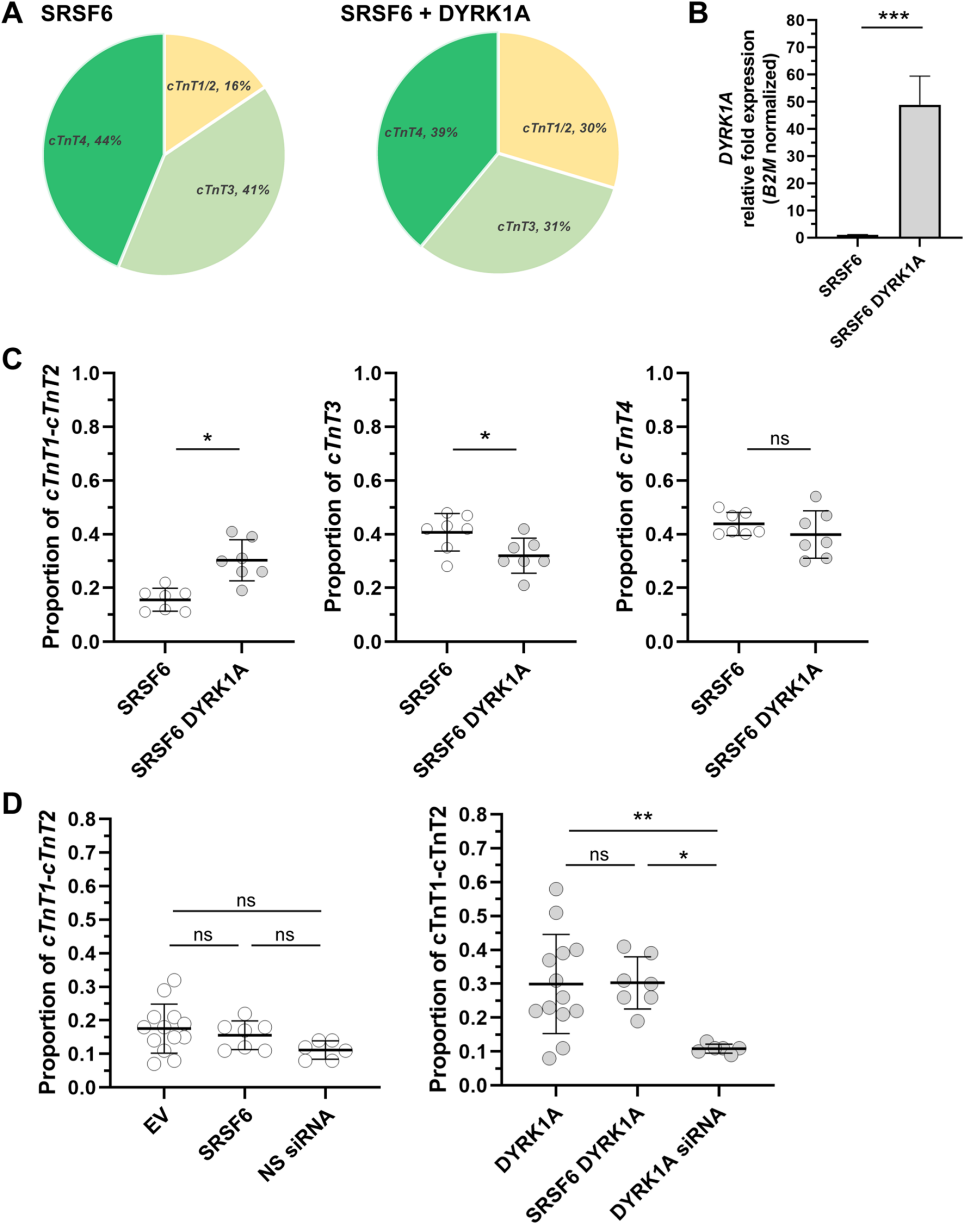
Impact of *DYRK1A* overexpression on the relative abundance of *TNNT2* transcript variants in iPSC cardiomyocytes transfected with *SRSF6*. **A** Relative abundance of *TNNT2* transcript variants in cells transfected with an *SRSF6-HA* (SRSF6) or co-transfected with *DYRK1A-GFP* and *SRSF6* expression constructs (SRSF6-DYRK1A). **B** Relative *DYRK1A* mRNA fold expression in transfected cells. **C** Relative abundance of *cTnT1* and *cTnT2* (left), *cTnT3* (center), and *cTnT4* (right) transcript variants. Horizontal bars show the mean ± SD from three independent transfections per condition, with 2–3 determinations performed in triplicate. ***P < 0.001, *P < 0.05, *ns* not significant, Student’s *t* test. **D** Impact of *SRSF6* overexpression on the relative abundance of *cTnT1* and *cTnT2* in cells with basal levels of *DYRK1A* (left), or overexpressing *DYRK1A* (right). **P < 0.01, *P < 0.05, *ns* not significant. One-way ANOVA (Tukey’s test)

Next, the expression of *TNNT2* fetal and adult splicing variants was assessed in iPSC cardiomyocytes co-transfected with *SRSF6* and *DYRK1A* expression constructs (Fig. 4A–C). Concomitant *DYRK1A-SRSF6* overexpression caused a ~ 93% increase in the proportion of *cTnT1* and *cTnT2* (*cTnT1/2*_SRSF6-*DYRK1A*_: 0.31 ± 0.08, *cTnT1/2*_SRSF6_: 0.16 ± 0.04) and a ~ 56% decrease in *cTnT3* in comparison to cells transfected with *SRSF6* only (*cTnT3*_SRSF6-*DYRK1A*_: 0.32 ± 0.06, *cTnT3*_SRSF6_: 0.41 ± 0.07). Similar comparisons revealed no significant changes in the proportion of *cTnT4* splicing variants (*cTnT4*_SRSF6-DYRK1A_: 0.40 ± 0.09, *cTnT4*_SRSF6_: 0.44 ± 0.04) (Fig. 4C). Finally, the impact of *SRSF6* overexpression in the proportion of *cTnT1* and *cTnT2* variants was assessed in iPSC cardiomyocytes either expressing basal or increased levels of *DYRK1A* mRNA. Comparisons revealed no significant changes in the proportion of *TNNT2* fetal splicing variants in *SRSF6* transfected iPSC cardiomyocytes and non-*SRSF6* transfected cells (Fig. 4D).

### DYRK1A Over-Expression and Sensitivity to Daunorubicin

**Fig. 5:**
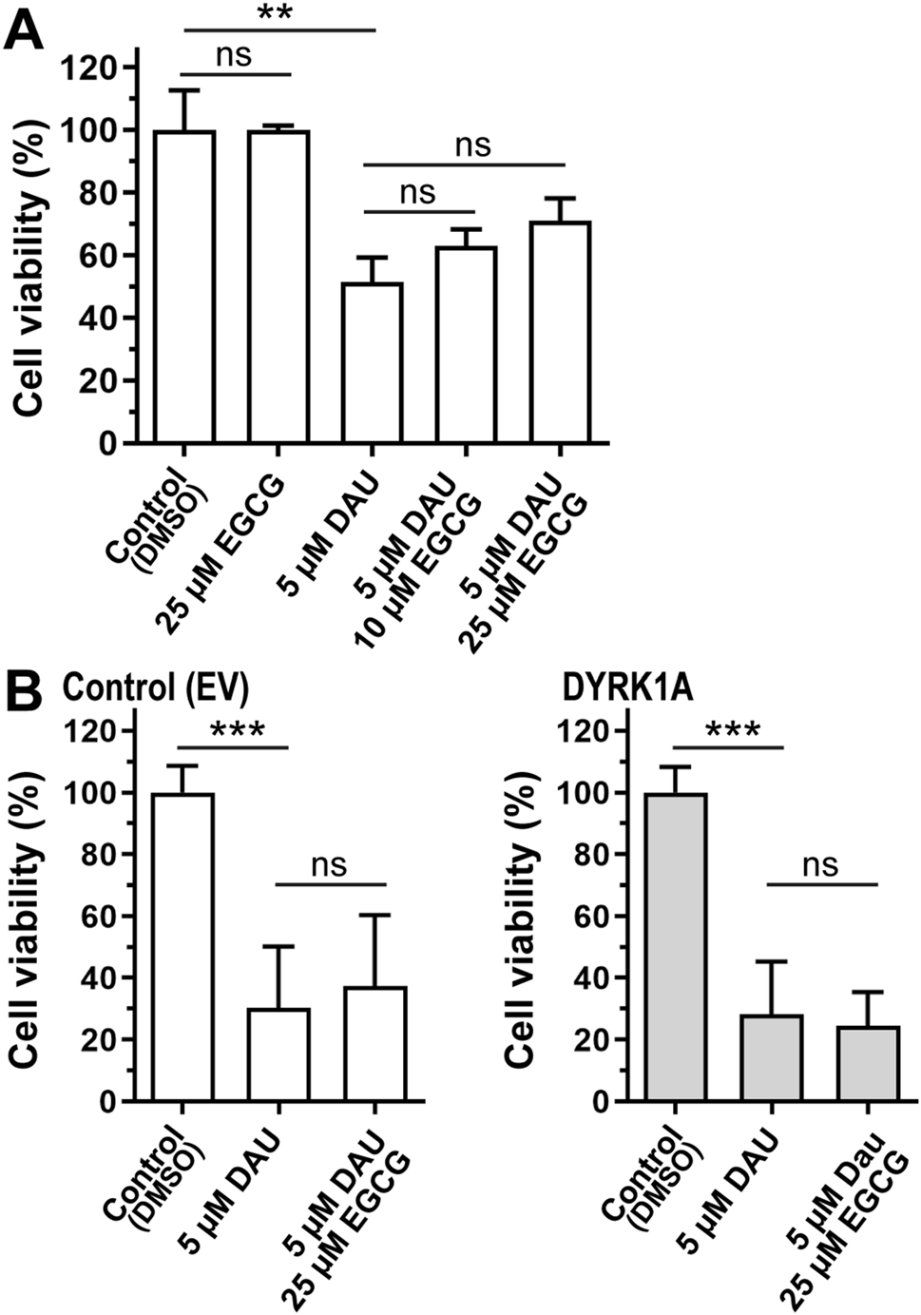
Impact of *DYRK1A* expression and drug treatments on the viability of iPSC cardiomyocytes. **A** Cellular viability after incubations with daunorubicin (DAU) and epigallocatechin gallate (EGCG). **B** Viability in controls and *DYRK1A-*overexpressing cells. Mean ± SD from two determinations performed in triplicates. ***P < 0.001, **P < 0.01, *ns* not significant, One-way ANOVA (Tukey’s test) and Student’s *t* test

We reasoned that variable DYRK1A expression may impact the sensitivity of iPSC cardiomyocytes to DAU. First, we examined whether treatment with epigallocatechin gallate (EGCG), a recognized DYRK1A inhibitor, modifies the cytotoxicity of the anthracycline daunorubicin (DAU) in iPSC cardiomyocytes. Cell viability was not significantly affected when EGCG was present during incubations with DAU (5 µM, 14 h) in cells with basal levels of *DYRK1A* expression (Fig. 5A). Increased *DYRK1A* expression did not modify the cytotoxicity of DAU (EV_5 µM DAU_: 30.41 ± 19.73%, DYRK1A_5 µM DAU_: 28.28 ± 17.04%) (Fig. 5B). In the context of *DYRK1A* over-expression, incubations with EGCG did not modify the cytotoxicity of DAU (EV_5 µM DAU_: 30.41 ± 19.73%, EV_5 µM DAU+ 25 µM EGCG_: 37.24 ± 23.10%; DYRK1A_5 µM DAU_: 28.28 ± 17.04%, DYRK1A _5 µM DAU+ 25 µM EGCG_: 24.41 ± 10.99%) (Fig. 5B).

**Fig. 6:**
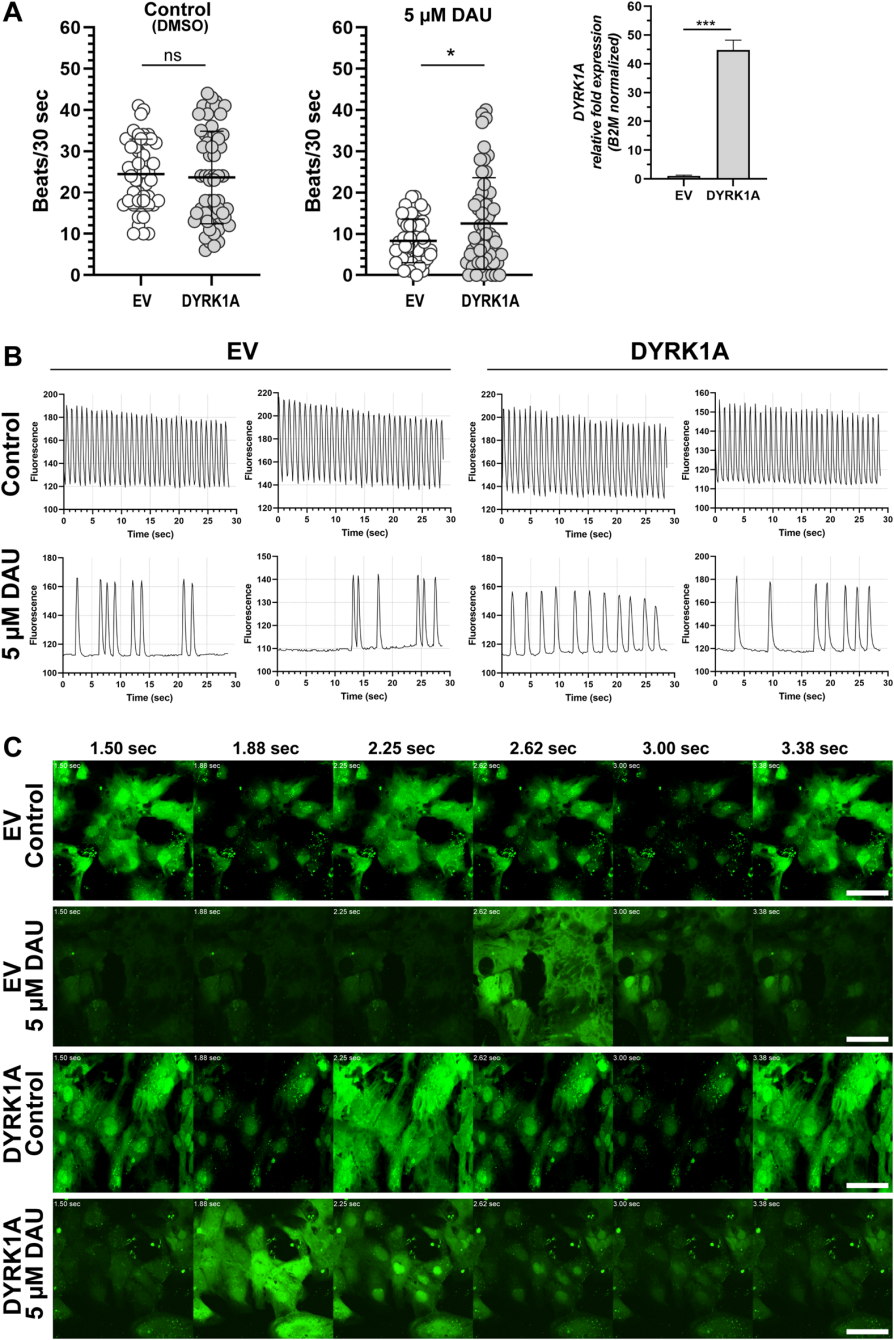
Analysis of iPSC cardiomyocytes contractility. **A** Beating rate in cardiomyocytes overexpressing *DYRK1A* in control conditions (left) and after exposure to daunorubicin (DAU) (middle). Right: relative *DYRK1A* fold expression in transfected cells. Each point represents individual measurements in a region of interest (ROI) (*n* = 45–55 ROIs per condition). Each bar represents the mean ± SD of three measurements performed in triplicates for each condition. *P < 0.05, *ns* not significant, Student’s *t* test. **B** Representative fluorograms showing beating patterns from controls and treated cells. **C** Representative live-confocal microscopy images of iPSC cardiomyocytes showing changes in Fluo-4 AM intensity (green) over time. Scale bar: 50 µm

Next, we examined whether *DYRK1A* overexpression in combination with DAU treatment modifies the beating frequency of cardiomyocytes. Beating frequency was determined by live-cell imaging with a calcium-sensitive fluorescent dye (Fig. 6, Videos 1, 2, 3 and 4). Assays were performed at day 14 post initial plating and 48 h post-transfection. Under these conditions, iPSC cardiomyocytes formed a synchronized contracting syncytium (Videos 1 and 2). In control conditions, *DYRK1A* overexpression (*DYRK1A* mRNA expression > 45-fold) did not modify the beating frequency of cardiomyocytes (EV: 24.5 ± 8.4 beats/30 s, DYRK1A: 23.7 ± 11.2 beats/30 s). In cardiomyocytes over-expressing *DYRK1A*, incubation with DAU decreased beating frequency by ~ 47% vs a ~ 66% decrease in controls transfected with empty vector (EV_5 µM DAU_: 8.3 ± 5.3, DYRK1A_5 µM DAU_: 12.5 ± 11.1) (Fig. 6A, B, and Videos 3 and 4).

## Discussion

This study examined *DYRK1A*-S*RSF6*-*TNNT2* interplays in beating iPSC cardiomyocytes. Increased *DYRK1A* expression in cardiomyocytes resulted in a **~** 58% increase in the endogenous relative abundance of the fetal variants *cTnT1* and *cTnT2*, and a decrease of **~** 27% in the abundance of the *cTnT3* variant. The relative abundance of the *cTnT4* variant remained unchanged (Figs. 1, 4). In line with our observations, a previous study in immortalized human embryonic kidney HEK-293 cells showed that *DYRK1A* overexpression increased the retention of exon 5 in PCR products derived from the splicing of an artificial *TNNT2* “mini-gene”. In the same study, the authors showed that increased cardiac *DYRK1A* expression in Ts65Dn mice, a model of Down syndrome, delayed the exclusion of exon 5 during the neonatal period [15]. Results from our previous analysis of myocardial tissue samples from donors with Down syndrome suggest that the higher relative abundance of the fetal troponin variants *cTnT1* and *cTnT2* seems to persist during adulthood (i.e., **~** 50% increase in comparison to myocardial samples from subjects without Down syndrome) [16].

DYRK1A modulates the splicing activity of SR proteins mainly through selective phosphorylation of their proline-rich domain, and also the RS1 domain [23]. Phosphorylation of SRSF6 by DYRK1A promotes the inclusion of exon 5 into *TNNT2* transcripts, and increased levels of phosphorylated SRSF6 were detected in myocardium from subjects with Down syndrome [15, 16]. Because detection of post-translational modifications is usually challenging and requires large amounts of material, we examined the phosphorylation status of HA-tagged SRSF6. In the context of *DYRK1A* over-expression, SRSF6 phosphorylation was **~** 25–65% higher in SRSF6-HA immunoprecipitated samples in comparison to controls (Fig. 3). In line with our observations, Yin et al. reported a 20% decrease in the phosphorylation of SRSF6 by DYRK1A after deletion of the proline-rich and RS1 domains of SRSF6 [23]. The overexpression of SRSF6 alone did not impact the relative abundance of *TNNT2* transcripts variants (Fig. 4D). Thus, the observed shift in *TNNT2* transcripts variants in the context of elevated *DYRK1A* expression may be mediated by an increase in SRSF6 phosphorylation by DYRK1A.

Some pediatric patients with acute myeloid leukemia and Down syndrome develop anthracycline-related cardiotoxicity [24, 25]. It is unclear why some patients treated with anthracyclines develop cardiotoxicity whereas others identically treated do not. The expression of myocardial *DYRK1A* in diploid and trisomic myocardium is variable. We documented higher expression of fetal *TNNT2* variants in myocardial tissue from donors with Down syndrome in comparison to samples from donors without Down syndrome [16]. We reasoned that increases in the relative abundance of fetal *TNNT2* transcript variants resulting from *DYRK1A* overexpression may render cardiomyocytes more sensitive to DAU cytotoxicity. The cytotoxic activity of DAU was similar between iPSC cardiomyocytes with basal and high levels of *DYRK1A* expression (Fig. 5). Cardiomyocytes expressing basal levels of *DYRK1A* showed a non-statistically significant trend towards increased cell viability after concomitant incubations with the DYRK1A inhibitor ECGC and DAU (Fig. 5A, B). The inhibition of DYRK1A by ECGC has been examined in preclinical studies with the aim of improving cognitive functions in Down syndrome [26–28]. ECGC has antioxidant properties and target other proteins besides DYRK1A [29, 30]. For example, the ECGC derivative Y6 decreases the expression of the anthracycline reductase CBR1 and the synthesis of daunorubicinol which in turn reduces DAU cardiotoxicity [31]. Our results suggest that in the context of *DYRK1A* overexpression, ECGC does not protect cardiomyocytes against DAU cytotoxicity (Fig. 5B). Cardiac myofibrils containing fetal troponin isoforms are more sensitive to Ca^2+^ which influences the contractile properties of myocardium [5, 32, 33]. iPSC cardiomyocytes with basal or increased levels of *DYRK1A* exhibited similar beating frequencies as determined by confocal live-cell imaging with a Ca^2+^ sensitive dye (Fig. 6A). Incubations with DAU reduced the beating frequency of cardiomyocytes by **~** 56%. The overexpression of *DYRK1A* ameliorated the impact of DAU on beating frequency (Fig. 6 and Videos 1, 2, 3 and 4). Increased expression of the fetal isoforms cTnT1/cTnT2 has been detected in failing hearts [7, 34]. Evidence suggests that the expression of fetal cTnT isoforms in the failing heart could represent a cardioprotective response [35].

One of the main limitations of this study is that iPSC cardiomyocytes were transiently transfected, and only a fraction of the cell population showed increased *DYRK1A* expression. Thus, it is possible to speculate that the observed differences in *TNNT2* splicing and beating in response to DAU could be more pronounced in cultures with higher efficiencies of cellular transfection. Thus, it would be of interest to further examine whether alterations in *DYRK1A-TNNT2* expression in vivo or in more nuanced cellular scenarios such as iPSC cardiomyocytes with trisomy 21 or cells with various degrees of stable *DYRK1A* over-expression modify Ca^2+^ handling and beating in response to cardiotoxic insults including anthracyclines. Another limitation is that we did not examine absolute amounts of each *TNNT2* mRNA splicing variant in cardiomyocytes. Our methodology is similar to the one used by Lu and Yin for the examination of *TNNT2* splicing patterns, and our observations further support the notion that increased *DYRK1A* expression impacts the relative expression of *TNNT2* splicing variants [15].

Although iPSC-derived cardiomyocytes have limited relevance to model the biology of the adult heart, they provide a more suitable platform for the study of drug metabolism and disposition than transformed cell lines with abnormal karyotypes and deregulated transcriptional networks [1]. A recent systematic study showing major limitations of cardiac cells revealed that gene expression in iPSC-derived cardiomyocytes is more similar to adult cardiac tissue compared to the cardiac cell lines AC16, H9C2 and HL-1. Nevertheless, the expression of elements of the contractile machinery was decreased in human AC16 and iPSC-derived cardiomyocytes compared to healthy adult cardiac samples [36]. In this context, the translational relevance of *DYRK1A* expression on *TNNT2* splicing and anthracycline toxicity remains to be determined. Points that merit further consideration are (1) analysis of beating frequency and sensitivity to DAU in cells overexpressing fetal cTnT isoforms, (2) evaluation of the impact of alternative concentrations of DAU and other anthracyclines, (3) study of the contribution of DYRK1A phosphorylation to DAU response and SRSF6 activity in iPSC cardiomyocytes, and (4) quantitative analysis of *TNNT2* mRNA splicing variants and other markers of cytotoxicity (e.g., LDH activity) in cardiomyocytes exposed to anthracyclines.

In conclusion, this study contributes new information on the expression of endogenous *TNNT2* transcript variants and SRSF6 phosphorylation in the context of altered *DYRK1A* expression in a relevant model of human beating cardiomyocytes. This work lays the foundation to further investigate the contribution of variable *DYRK1A-TNNT2* expression to drug-induced cardiotoxicity in human cardiomyocytes and in vivo models.

## Supplementary Information

Below is the link to the electronic supplementary material.

Supplementary file1 (DOCX 1491 kb)

Supplementary file2 (ZIP 24238 kb)

## Data Availability

The datasets generated for this study are available from the corresponding author upon request.
