# Supplementary material for: Impact of DYRK1A Expression on TNNT2 Splicing and Daunorubicin Toxicity in Human iPSC-Derived Cardiomyocytes
Source: Cardiovasc Toxicol. 2022 May 21;22(8):701–12. doi: 10.1007/s12012-022-09746-6 (PMC9236996; doi:10.1007/s12012-022-09746-6)
Supplement: Supplementary file 1 — Supplementary file1 (DOCX 1491 kb) [file 12012_2022_9746_MOESM1_ESM.docx]

**Supplemental Information**

**Impact of *DYRK1A* expression on *TNNT2* splicing and daunorubicin toxicity in human iPSC-derived cardiomyocytes.**

Cejas RB^1^, Tamaño-Blanco M^1^, Fontecha JE^2^, and Blanco JG^1, *^

^1^ Department of Pharmaceutical Sciences, School of Pharmacy and Pharmaceutical Sciences, The State University of New York at Buffalo, Buffalo, NY 14214, USA

^2^ Group for Applied Mathematical Modeling and Analytics (GAMMA), Industrial and Systems Engineering, University at Buffalo, Buffalo, NY, USA

* Corresponding author at: Department of Pharmaceutical Sciences, University at Buffalo, The State University of New York, 470 Pharmacy building, Buffalo, NY 14214 – 8033, USA

Phone: 716 - 645 - 4820

Fax: 716 - 829 - 6569

E-mail: jgblanco@jgblanco@buffalo.edubuffalo.edu

**Table S1.** qRT-PCR primers.

|  | **Primer forward sequence 5’🡪3’** | **Primer reverse sequence 5’🡪3’** |  |
| --- | --- | --- | --- |
| *cTnT1/cTnT2* | GGAGGACTGGAGAGAGGAC | CACCAAGTTGGGCATGAACG |  |
| *cTnT3* | CTGTTGAAGAGCAGGAGGAG | CCGACGTCTCTCGATCCTG |  |
| *cTnT4* | AGCAGGAAGAGCAGGAGGAG | CCGACGTCTCTCGATCCTG |  |
| *DYRK1A* | GGACAGGTTGTAAAGGCATATG | GCGTTTCAAATGCACTATGTAG |  |
| *B2M* | TGAAGCTGACAGCATTCGG | CTGCTGGATGACGTGAGTAAA |  |


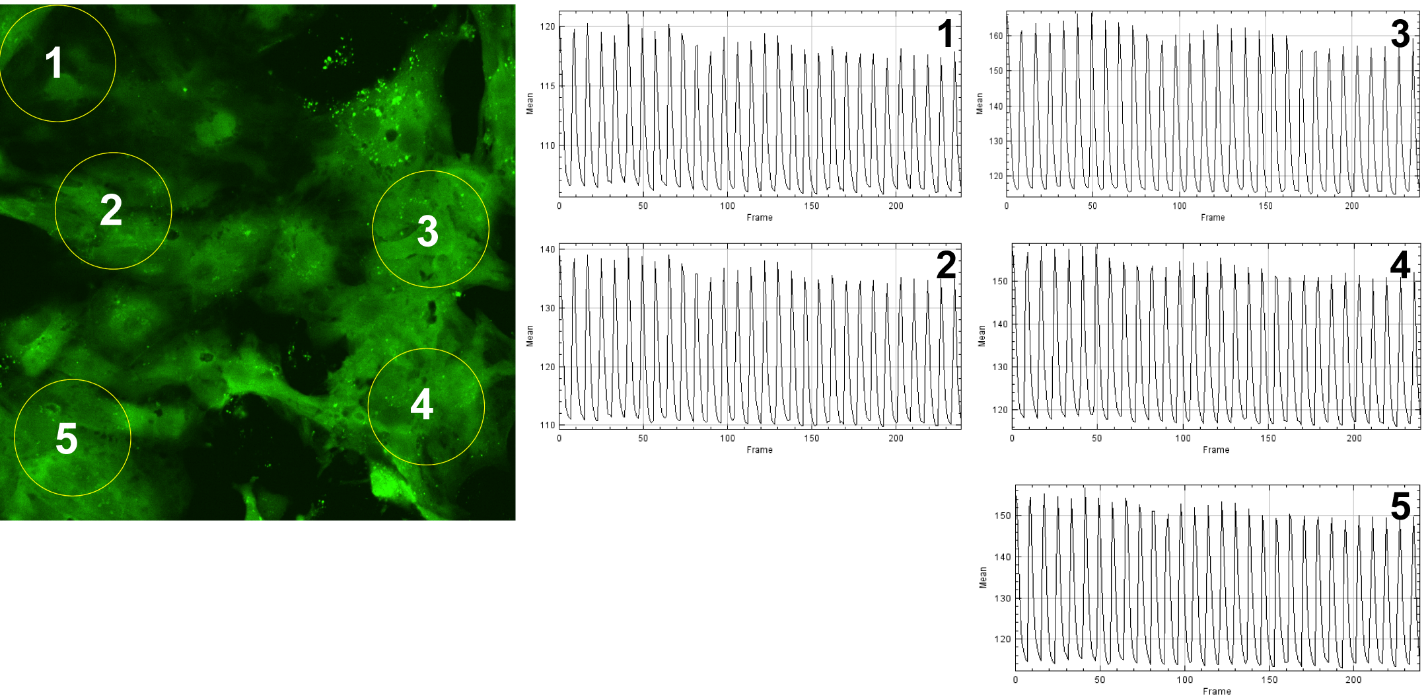


**Figure S1.** **Cellular regions of interest for quantitative image analysis.** Regions of interest (ROIs) including groups of beating iPSC cardiomyocytes (yellow circles, 1 to 5) were created by manual selection (left). Middle and right panels: fluorograms obtained from each ROI. Note that beating profiles are similar for all ROIs within the image stack.

**
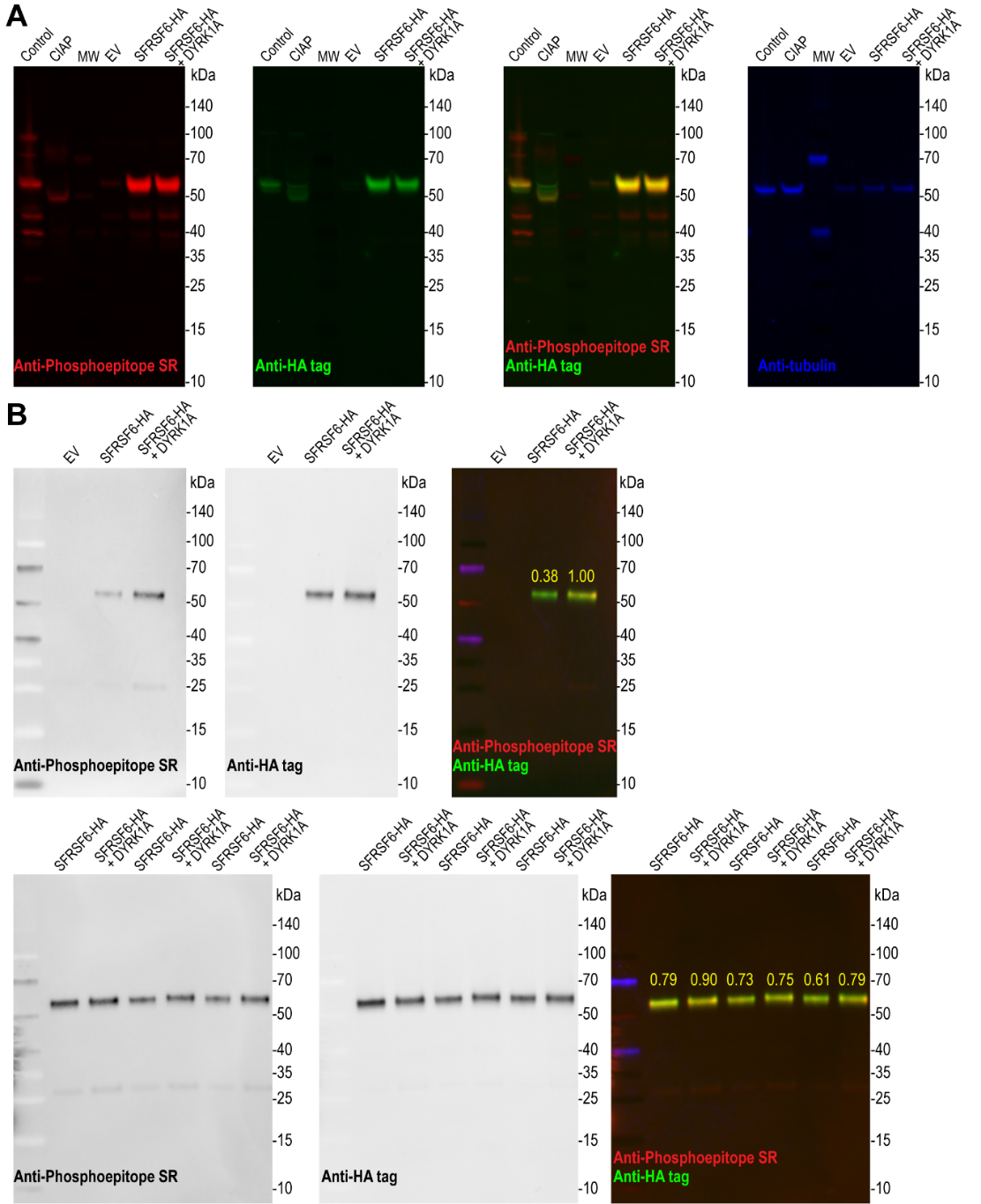
**

**Figure S2. Full-size immunoblots of cropped regions shown in Figure 3.** Phospho-terminals in SR proteins (red) and HA-tag (green) detected by immunoblotting with specific antibodies. Phospho SR and HA-tag signals are shown merged to visualize phosphorylated SRSF6 (yellow). **A.** Lanes 1 and 2: AC16 cardiomyocytes overexpressing SRSF6-HA before (Control) and after treatment with calf intestine alkaline phosphatase (CIAP). Lane 3: molecular weight marker (MW). Lanes 4 to 6: iPSC cardiomyocytes transfected with an empty vector (EV), a *SRSF6-HA* construct (SRSF6-HA) or co-transfected with *DYRK1A* and *SRSF6-HA* (SRSF6-HA DYRK1A). β-tubulin was assayed as loading control (blue). **B.** HA-immunoprecipitated samples from iPSC transfected cardiomyocytes. The phospho SR/HA-tag signal ratio was quantified by densitometric analysis and values are denoted in yellow above each protein band.

**Video 1.** Contractibility of iPSC cardiomyocytes transfected with an empty vector (EV) after exposure to DMSO vehicle (control). Cells were stained with Fluo-4 AM (green). The time lapse stack is composed by 240 frames imaged at a frame interval of 0.125 sec/frame in a total interval of 30 sec.

**Video 2.** Contractibility of iPSC cardiomyocytes overexpressing *DYRK1A* after exposure to DMSO vehicle (control). Cells were stained with Fluo-4 AM (green). The time lapse stack is composed by 240 frames imaged at a frame interval of 0.125 sec/frame in a total interval of 30 sec.

**Video 3.** Contractibility of iPSC cardiomyocytes transfected with an empty vector (EV) after exposure to 5 µM Daunorubicin for 14h. Cells were stained with Fluo-4 AM (green). The time lapse stack is composed by 240 frames imaged at a frame interval of 0.125 sec/frame in a total interval of 30 sec.

**Video 4.** Contractibility of iPSC cardiomyocytes overexpressing *DYRK1A* after exposure to 5 µM Daunorubicin for 14h. Cells were stained with Fluo-4 AM (green). The time lapse stack is composed by 240 frames imaged at a frame interval of 0.125 sec/frame in a total interval of 30 sec.
